# Supplementary material for: Assessment of Treatment Response after Pressurized Intra-Peritoneal Aerosol Chemotherapy (PIPAC) for Appendiceal Peritoneal Metastases
Source: Cancers (Basel). 2022 Oct 12;14(20):4998. doi: 10.3390/cancers14204998 (PMC9599491; doi:10.3390/cancers14204998)
Supplement: Supplementary file 1 [file cancers-14-04998-s001.zip › cancers-1882553-supplementary.pdf]

Online appendix PP Cohort Cox regression analysis for OS

Table S1. Univariate analysis.

Variables		beta	HR (95% CI)	Wald test	P value
**Gender**	Male	0.56	1	1.2	0.28
	Female		1.7 (0.64-4.8)		
	Age	-0.0053	0.99 (0.96-1)	0.07	0.8
	BMI	-0.056	0.95 (0.83-1.1)	0.64	0.42
ASA score	1	-1	1	3.3	0.19
	2		-1.4 (0.1-1.3) "3.3"		
	3		-1.4 (0.044-1.4)		
ECOG	ECOG0	0.59	1	4.8	0.089
	ECOG1		1.5 (0.45-7.3)		
	ECOG2		1.5 (1.2-17)		
Pathology	No	-0.16		0.05	0.83
	Yes		0.85 (0.19-3.7)		
Histology	1	0.54		1.3	0.45
	2		-0.051 (0.47-9.6)		
	3		-0.051 (0.24-3.8)		
RAS	No				
	Yes	-1.2	0.32 (0.077-1.3)	2.6	0.11
Previsous CRS + HIPEC	No				
	Yes	1.2	3.2 (0.7-15)	2.2	0.14
CRS	No				
	Yes	-0.26	0.77 (0.27-2.2)	0.24	0.63
Previous chemotherapy	No				
	Yes	0.52	1.7 (0.38-7.4)	0.46	0.5
Regimen previous chemotherapy	oxaliplatine based	0.36		1.9	0.6
	irinotecan based		-1.1 (0.38-5.4)		
	oxiri based		-1.1 (0.042-2.7)		
	other		-1.1 (0.22-15)		
Previsous biological treatment	No				
	Yes	0.56	1.7 (0.6-5.1)	1	0.31
Cycles before PIPAC		-0.064	0.94 (0.73-1.2)	0.25	0.62
Second chemotherapy line	No				
	Yes	-0.64	0.53 (0.19-1.5)	1.4	0.23
Third chemotherapy line	No				
	Yes	-19	4.4e-09 (0-Inf)	0	1
Number cycles third chemo	No				
	Yes	-19	4e-09 (0-Inf)	0	1
Total cycles		-0.29	0.75 (0.6-0.93)	6.6	0.01
Total cycle	≤12				
	>12	-21	9.8e-10 (0-Inf)	0	1
Symptomes prePIPAC	No				
	Yes	-0.49	0.62 (0.24-1.6)	0.98	0.32
Chemo PIPAC	No				
	Yes	0.41	1.5 (0.52-4.4)	0.57	0.45
Avastin PIPAC	No				
	Yes	0.85	2.4 (0.84-6.6)	2.7	0.1
Cycle PIPAC		-0.12	0.89 (0.7-1.1)	0.89	0.35
CEA prePIPAC		0.015	1 (1-1)	4.1	0.043
Ca19.9 prePIPAC		0.0051	1 (1-1)	2.6	0.11
Ca125 prePIPAC		0.028	1 (0.95-1.1)	0.48	0.49
Creatinin prePIPAC		-0.0086	0.99 (0.97-1)	0.43	0.51
Albumine prePIPAC		-0.23	0.8 (0.66-0.96)	6	0.014
Type chemo at PIPAC1	1				
	2	-0.12	1.9 (0.56-4.3)	3.1	0.21
	3		1.9 (0.72-58)		
PCI at PIPAC1		0.02	1 (0.97-1.1)	0.56	0.45
Ascites at PIPAC1		4.20E-04	1 (1-1)	5.5	0.019

Cytology at PIPAC1	No				
	Yes	-1.1	0.32 (0.065-1.6)	1.9	0.17
PIPAC Complications	No				
	Yes	-0.18	0.83 (0.27-2.6)	0.1	0.75
Radiological response at PIPAC3					
	Progression	1.3	3.7 (1-14)	4	0.045
	PRGS_PIPAC3	-0.96	0.38 (0.08-1.8)	1.4	0.23
	Cytology_PIPAC3	0.17	1.2 (0.23-6.2)	0.04	0.84
	Delta_PIC	0	1		
		1	2.8 (0.55-14)	1.5	0.22
	PCI_PIPAC3	0.057	1.1 (0.99-1.1)	2.6	0.11
	CEA_PIPAC3	-0.034	0.97 (0.9-1)	0.87	0.35
	Ca19.9_PIPAC3	0.00041	1 (1-1)	0.54	0.46
	Ca125_PIPAC3	-0.0012	1 (0.98-1)	0.01	0.91
	Creatinine_PIPAC3	0.00063	1 (0.98-1)	0	0.95
	Albumine_PIPAC3	0.054	1.1 (0.92-1.2)	0.56	0.45
	Symptoms_PIPAC3	0.56	1.7 (0.55-5.6)	0.88	0.35
Progression		0.21	1.2 (0.34-4.5)	0.1	0.75

Table S2. Multivariate analysis.

Variables	beta	HR (95% CI)	P value
Total cycle	0.52	1.93 (0.25,1.08)	0.079
CEA prePIPAC	1.00	1 (0.9,1.11)	0.984
Albumine prePIPAC	0.90	1.11 (0.59,1.39)	0.643
Ascites at PIPAC1	1.00	1 (1,1.01)	0.188
Radiological progression at PIPAC3	1.82	0.55 (0.08,39.79)	0.704
